# *oxi-1* is required for chemotaxis to odorants sensed by AWA but not AWC neurons

**DOI:** 10.17912/micropub.biology.001282

**Published:** 2024-08-19

**Authors:** Muiz Rana, Jennifer Kowalski

**Affiliations:** 1 Biological Sciences, Butler University, Indiana, United States of America

## Abstract

This study examines the role of the *oxi-1 UBE3B* gene in chemotaxis of *C. elegans* to volatile odorants. Compared to wild type worms, *oxi-1* mutants showed no difference in chemotaxis to the AWC-specific odorant, isoamyl alcohol but a significant decrease in chemotaxis compared to *odr-7* mutants. Both *oxi-1* and *odr-7* mutants exhibited significant decreases in chemotaxis to AWA-specific odorants, pyrazine and diacetyl. For thiazole, which is sensed by both AWA and AWC neurons, only *odr-7* mutants showed significantly decreased chemotaxis. These data demonstrate *oxi-1* is required for chemotaxis to AWA- but not AWC-specific odorants, the mechanisms of which should be investigated.

**Figure 1. oxi-1 mutants show decreased chemotaxis towards the AWA-specific odorants, pyrazine and diacetyl, similar to odr-7 mutants:**
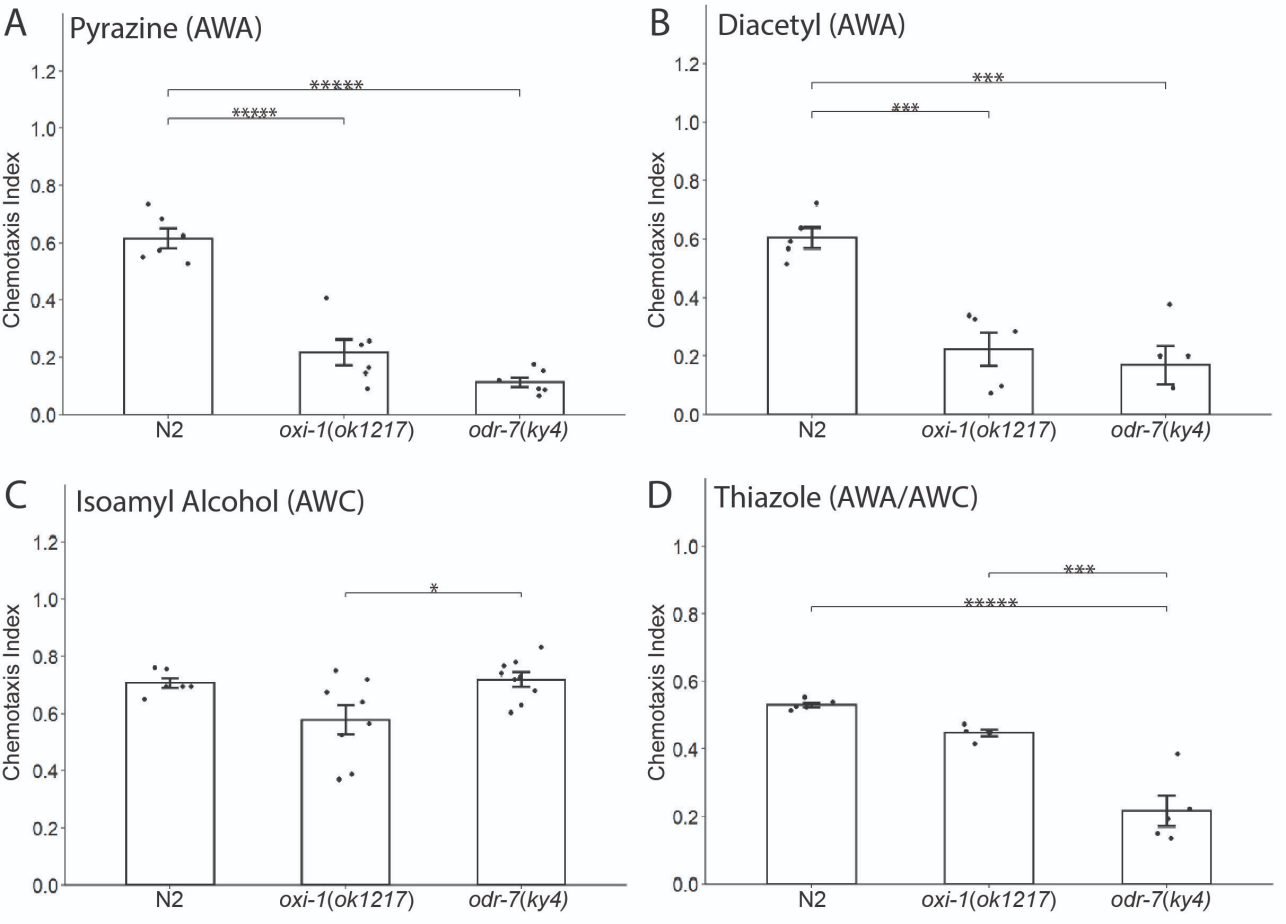
Wild type (N2), *oxi-1* ( *ok1217* ), and *odr-7(ky4* ) mutant worms at the L4 and young adult stage were tested in chemotaxis assays towards the following odorants: **(A)** pyrazine, **(B)** diacetyl, **(C)** isoamyl alcohol, and ( **D)** thiazole. *oxi-1* and *odr-7* mutants showed a statistically significant decrease in chemotaxis to both AWA-specific odorants, pyrazine and diacetyl, and modest but insignificant decreases in chemotaxis to the AWC-specific odorant, isoamyl alcohol, and to thiazole, which is sensed by both AWA and AWC neurons (Bargmann, 2006). Bars indicate the means of the datasets, points indicate the average chemotaxis index per experiment (n = 5-9 experiments per odorant), and error bars indicate the standard deviation of the means. One-way ANOVA with Tukey's post hoc test was used for statistical analysis; * *p* <0.05, *** *p* <0.001, ***** *p* <0.00001.

## Description

The olfactory system, integral to the nervous system, detects airborne chemicals. In humans, this sense is critical for taste, detection of dangers, and many other everyday tasks (Spielman, 1998) . *UBE3B,* a gene encoding a HECT domain E3 ubiquitin ligase that is crucial for human cognitive function, is widely expressed throughout the brain, including in the olfactory bulb (Ambroszkiewicz et al., 2020), suggesting its potential relevance to olfactory function. Loss of function mutations in *UBE3B* can lead to diseases such as Kaufman oculocerebrofacial syndrome , an intellectual disability syndrome which can affect craniofacial features, including the shape and orientation of the nostrils (MedLine Plus, 2017). Previous work has shown mammalian UBE3B regulates synapse number and hippocampal function through effects on dendritic spines, potentially due to its ability to ubiquitinate and downregulate the catalytic subunit of calcineurin, Ppp3cc (Ambrozkiewicz et al., 2021) . However, whether and how *UBE3B* regulates olfactory function is unknown.

It is hard to study *UBE3B* in humans with billions of neurons, so *Caenorhabditis elegans* roundworms with simpler nervous systems of only 302 neurons are used. The protein sequences of human *UBE3B* and worm *oxi-1* are 37% identical and 58% similar, and *oxi-1* appears orthologous to human *UBE3B* (Basel-Vanagaite et al., 2012) . Thus, investigations into *oxi-1* function may provide useful insight into conserved roles of *UBE3B* . In *C. elegans* , *oxi-1* was identified as an oxidative stress-responsive gene (Yanase and Ishi, 1999) and is required for protein ubiquitination under low level oxidative stress, as well as for locomotion under both normal and oxidative stress conditions (Basel-Vanagaite et al. *,* 2012; Wei and Kowalski, 2018). Expression of *oxi-1* has been reported in numerous motor, sensory, and interneurons in *C. elegans,* including AWA and AWC (Taylor et al *.,* 2021). However, it is unknown what role *oxi-1* plays in the chemosensory function of the worms. This raises the question: Is *oxi-1* required for chemotaxis to volatile odorants sensed by these neurons?

To address this question, *oxi-1* ( *ok1217* ) deletion mutant worms were tested in chemotaxis assays to both AWA-specific ( pyrazine , diacetyl ) and AWC-specific ( isoamyl alcohol ) odorants, as well as thiazole , an odorant sensed by both AWA and AWC neurons. In these assays, an odorant is spotted on one end of a 10 cm plate and the counter-attractant on the other side. Worms are then spotted across the middle of the plate and are free to choose which side of the plate to crawl to in the time span of 1 hour. At the end of that hour, the worms are counted and the chemotaxis index is calculated (Bargmann et al., 1993; Wang et al., 2016) . In our studies, *oxi-1* ( *ok1217* ) mutants were compared to both wild type worms and to *odr-7* ( *ky4* ) mutant worms, which are defective in chemotaxis to AWC-specific odorants (Bargmann et al., 1993) .

Results of experiments testing attraction to the AWA neuron-specific odorants, pyrazine and diacetyl , revealed statistically significant chemotaxis defects in both *oxi-1* and *odr-7* mutants. In experiments with pyrazine , *oxi-1* mutants showed a 65% decrease and *odr-7* mutants an 85% decrease in chemotaxis indices compared to N2 wild type worms ( Figure 1A ). Both *oxi-1* and *odr-7* mutants also showed reductions (65% and 72%, respectively) in their chemotaxis towards diacetyl compared to N2 wild type worms ( Figure 1B ). In contrast, experiments measuring chemotaxis to the AWC neuron-specific attractant, isoamyl alcohol , indicated *oxi-1* mutants have a modest but statistically insignificant 19% decrease in chemotaxis compared to N2 wild type worms, whereas *odr-7* mutants experienced a small but also insignificant 2% increase in chemotaxis ( Figure 1C ). Additionally, when comparing *oxi-1* to *odr-7* mutant animals, *oxi-1* mutants had a significant difference of 20% less chemotaxis towards isoamyl alcohol than *odr-7* mutants ( Figure 1C ). Finally, in experiments testing chemotaxis to the AWA and AWC neuron-specific attractant, thiazole , *oxi-1* mutants showed a non-significant 15% decrease in chemotaxis compared to N2 wild type worms, whereas *odr-7* mutant worms showed a statistically significant 59% decrease in chemotaxis. When comparing *oxi-1* to *odr-7* , *oxi-1* animals had a significant 50% increase in chemotaxis index over *odr-7* mutant worms ( Figure 1D ).

The AWA and AWC olfactory neurons in *C. elegans* play fundamental roles in chemotaxis behavior by detecting volatile odors (Bargmann, 2006; Ferkey et al., 2021) . Through the detection of specific compounds, such as diacetyl , pyrazine , thiazole , benzaldehyde , and isoamyl alcohol , these neurons facilitate the organism's ability to navigate its surroundings effectively. The distinct sensitivities of AWA and AWC neurons to different odors highlight their specialization in odor detection. This specialization highlights the importance of these sensory neurons in guiding the behaviors of *C. elegans* , emphasizing their significance in sensory processing and environmental adaptation. Here, we found that there was a significant decrease in chemotaxis with *oxi-1* using AWA-specific odorants ( pyrazine and diacetyl ), but not when using AWC-specific ( isoamyl alcohol ) or an odorant sensed by both AWA and AWC neurons ( thiazole ). This suggests a specific requirement for *oxi-1* , like *odr-7* , in the circuit for detection of AWA- but not AWC-specific odorants.

*odr-7* and *oxi-1* in *C. elegans* may exhibit different isoamyl alcohol and thiazole phenotypes due to their distinct roles and mechanisms in different neurons. *odr-7* is a nuclear receptor expressed in the AWA olfactory neurons of *C. elegans* (Sengupta et al., 1994) . *odr-7* regulates the development and function of these neurons by acting as a transcription factor that is essential for AWA cell fate specification (Sengupta et al *.* , 1994), explaining why chemotaxis to all odorants sensed by AWA neurons ( pyrazine , diacetyl , and thiazole ) is affected in *odr-7* mutants. In contrast, although animals deficient in *oxi-1* exhibit similar deficits to pyrazine and diacetyl-induced chemotaxis, there is no significant reduction in chemotaxis to thiazole in these animals, suggesting a different mechanism of action. *UBE3B/ oxi-1* encodes an E3 ubiquitin ligase crucial for protein degradation in both *C. elegans* , where it is particularly required for protein degradation under oxidative stress (Basel-Vanagaite et al., 2012) , and in mammalian neurons, where several substrates have been identified (Ambrozkiewicz et al., 2021) . Thus, it is possible *oxi-1* may be acting in AWA neurons where its expression has been reported (Taylor et al, 2021) , to regulate the abundance of specific odorant receptors, such as the G protein-coupled receptor, ODR-10 (Sengupta et al. *,* 1996), or the TRPV ion channels OSM-9 or OCR-2 (Sokolchik et al. *,* 2005), or other signaling effectors important for sensing diacetyl and/or pyrazine but that are not involved in thiazole sensation (Ferkey et al., 2021) . By targeting proteins for degradation, *UBE3B/ oxi-1* may thus play a vital role in shaping neuronal circuits and maintaining their function (Ambrozkiewicz et al., 2020) . Mammalian UBE3B was shown to localize to mitochondria in cultured cells and to be regulated by calcium via interaction with calmodulin (Braganza et al. *,* 2017). Future experiments will be required to determine if these or other mechanisms of regulation govern OXI-1 function, to identify the substrates and sites of action of *oxi-1* in regulating these chemotaxis behaviors, as well as to elucidate whether these *oxi-1* -dependent responses change under oxidative or other stresses.

## Methods

***Strains and Strain Maintenance:*** Worms were maintained on standard NGM agar plates spotted with OP50 *E. coli* as described previously (Brenner, 1974) at 15°C prior to synchronization and testing in the chemotaxis assays. Strains used in this study are listed in the following table.

**Table:** 

**Strain**	**Genotype**
N2	wild type
CX4	*odr-7(ky4)*
RB1176	*oxi-1(ok1217)*

***Chemotaxis Assay:*** Three 10 cm assay plates for each strain to be tested ( N2 , *oxi-1 ,* and *odr-7* ) were prepared as described previously (Wang et al., 2016) . This assay set-up was developed to allow for shorter duration chemotaxis assays, to account for worms that migrated significantly to the odorant but may have been paralyzed at a distance from the attractant or counter-attractant spot, and ultimately to standardize the range of worms counted as “at the odorant” or “at the counter-attractant.” Briefly, plates were first marked with a dot at the center of the plate, through which a solid line was drawn, bifurcating the plate. Next, two dashed lines were drawn parallel to the center line, 2 cm away, one on each side of the centerline. Finally, one dot was placed on each half of the plate, 0.5 cm from the edge and in line with the center dot. One edge dot was labeled for ethanol , the other for the odorant to be tested. Labeled plates were left out to dry at room temperature for 2 days prior to the assay. Worms were synchronized by egg-laying for 1.5 hours, adults removed, and embryos grown 2 days at 25 ºC to L4/young adult stage. On the day of the experiment, the labeled assay plates were spotted with 10 µL 1 M sodium azide on both edge dots. Then, 10 µL ethanol (counter-attractant) or the odorant were spotted on the edge dots on opposite ends of the plate. Three plates for each strain were tested on each experimental day. Worms from the 6 cm plates were washed off and placed in 15 mL conical tubes. After removing excess liquid, worms were washed twice more with M9 and once with water, leaving approximately 100 µL and the worm pellet. [M9 was used as a simple wash solution to maintain physiological salt, pH, and osmotic conditions during food removal; we used M9 washes previously and obtained similar results to studies done with other wash solutions including water, HEPES-based buffers, and S basal medium (Wang et al., 2016; Ward, 1973; Bargmann et al., 1993; Kauffman et al., 2011) .] Following the washes, 20 µL of worms were placed on spots along the solid line. To prevent settling and to ensure that as many worms were collected in the set volume as possible, tubes were vigorously flicked with the finger prior to taking up the 20 µL. After all the worms were plated, corners of a Kimtech wipe were gently used to remove excess liquid that could result in inaccurate results. The worms were allowed to crawl around on the 10 cm plates for 1 hour at room temperature (~20-21ºC). After 1 hour, worms were counted above dashed lines near the odorant, near the counter-attractant, and in the sections in between. Only plates with 50+ worms were considered as acceptable results. Afterwards, the Chemotaxis Index was calculated by subtracting the worms at the counter-attractant from those at the odorant and dividing that value by the total number of worms on the 10 cm plate (number of worms at the odorant plus the number at the counter-attractant plus the number in the center of the plate). The calculation process was repeated for each of the nine 10 cm plates.

***Data Analysis:*** All assays were performed on 3 plates per strain per day and the mean for each strain computed by day. Thus, each assay was counted as a single independent replicate in the analysis. Only assays in which the wild type N2 worms had a Chemotaxis Index of 0.5 or greater were analyzed. Statistical significance of the means of the datasets was determined using a One-way ANOVA and Tukey's post hoc test in R Studio version 2022.02.0+443. All graphs were also produced in R studio.

## Reagents

***Materials:*** unseeded 10 cm NGM agar plates; pipettes and tips; stereomicroscope; ruler and markers

***Chemicals:*** 1 M sodium azide (Fisher Scientific #S-227); M9 buffer; diH _2_ 0; 95% ethanol (counter-attractant); odorants diluted in 95% ethanol (vol:vol) unless otherwise specified [ isoamyl alcohol (Sigma-Aldrich #M32658) at 1:1,000 dilution; diacetyl (Sigma-Aldrich #B85307) at 1:5,000 dilution; pyrazine (Sigma-Aldrich #P56003) at 1 mg/ml; thiazole (2,4,5-Trimethyl thiazole , Sigma-Aldrich #219185) at 1:1,000 dilution].
